# Clinical and molecular characterization of a large cohort of childhood onset hereditary spastic paraplegias

**DOI:** 10.1038/s41598-021-01635-2

**Published:** 2021-11-15

**Authors:** Gabriela Marchisio Giordani, Fabrício Diniz, Helena Fussiger, Carelis Gonzalez-Salazar, Karina Carvalho Donis, Fernando Freua, Roberta Paiva Magalhães Ortega, Julian Letícia de Freitas, Orlando Graziani Povoas Barsottini, Sergio Rosemberg, Fernando Kok, José Luiz Pedroso, Marcondes Cavalcante França, Jonas Alex Morales Saute

**Affiliations:** 1 Graduate Program in Medicine: Medical Sciences, Universidade Federal do Rio Grande do Sul, Porto Alegre, Brazil; 2 Neurogenetics, Clinical Research Center, Hospital de Clínicas de Porto Alegre (HCPA), Porto Alegre, Brazil; 3 Graduate Program in Medical Physiopathology, Universidade Estadual de Campinas (UNICAMP), Campinas, Brazil; 4 Medical Genetics Service, Hospital de Clínicas de Porto Alegre (HCPA), Ramiro Barcelos, 2350, Porto Alegre, 90035-903 Brazil; 5 Departamento de Neurologia, Hospital das Clínicas, Universidade de São Paulo, São Paulo, SP Brazil; 6 Irmandade da Santa Casa de Misericórdia de São Paulo, São Paulo, SP Brazil; 7 Department of Neurology, Ataxia Unit Universidade Federal de São Paulo, São Paulo, Brazil; 8 Faculdade de Ciências Médicas da Santa Casa de São Paulo, São Paulo, SP Brazil; 9 Department of Neurology, Faculdade de Ciências Médicas, Universidade Estadual de Campinas (UNICAMP), Campinas, Brazil; 10 Neurology Service, Hospital de Clínicas de Porto Alegre (HCPA), Porto Alegre, Brazil; 11 Internal Medicine Department, Faculdade de Medicina, Universidade Federal do Rio Grande do Sul, Porto Alegre, Brazil

**Keywords:** Diseases of the nervous system, Genetics of the nervous system, Neuroscience, Neurology, Neurological disorders, Genetics, Medical genetics, Sequencing

## Abstract

The present study aimed to characterize clinical and molecular data of a large cohort of subjects with childhood-onset hereditary spastic paraplegias (HSPs). A multicenter historical cohort was performed at five centers in Brazil, in which probands and affected relatives' data from consecutive families with childhood-onset HSP (onset < 12 years-old) were reviewed from 2011 to 2020. One hundred and six individuals (83 families) with suspicion of childhood-onset HSP were evaluated, being 68 (50 families) with solved genetic diagnosis, 6 (5 families) with candidate variants in HSP-related genes and 32 (28 families) with unsolved genetic diagnosis. The most common childhood-onset subtype was SPG4, 11/50 (22%) families with solved genetic diagnosis; followed by SPG3A, 8/50 (16%)*.* Missense pathogenic variants in *SPAST* were found in 54.5% of probands, favoring the association of this type of variant to childhood-onset SPG4. Survival curves to major handicap and cross-sectional *Spastic Paraplegia Rating Scale* progressions confirmed the slow neurological deterioration in SPG4 and SPG3A. Most common complicating features and twenty variants not previously described in HSP-related genes were reported. These results are fundamental to understand the molecular and clinical epidemiology of childhood-onset HSP, which might help on differential diagnosis, patient care and guiding future collaborative trials for these rare diseases.

## Introduction

Hereditary spastic paraplegias (HSP) are a group of heterogeneous genetic disorders characterized by the presence of progressive spastic hypertonia and muscle weakness, predominantly affecting the lower limbs, caused by the degeneration of corticospinal tract longest axons^1,2^. HSP are clinically classified as pure or complex forms. Individuals with pure HSP present an isolated pyramidal syndrome with or without vibratory sensation impairment or neurogenic bladder, whereas in individuals with complex HSP, the pyramidal syndrome is accompanied by additional neurological or systemic findings^3^. HSP are associated to > 83 genes or *loci* and to all patterns of inheritance, with ages at onset ranging from early infancy up to the eighth decade of life^2,4^.

Prevalence estimations of HSP in different geographical regions ranges from 2 to 10 per 100,000 individuals^5^. However, there is a lack of epidemiological data about HSP in Latin America, with a few studies suggesting SPG4 as the most frequent autosomal dominant and SPG11 as the most frequent autosomal recessive HSP subtype in Brazil^6^.

Little is known about clinical and molecular epidemiology of childhood onset HSPs^4,7–11^. Early reports in specific populations suggested that SPG3A was the most frequent subtype^7^; however, more recent studies in larger samples pointed SPG4 as the most common HSP across all age-groups^4^. Therefore, we aimed to characterize clinical and genetic data of a large cohort of HSPs with childhood onset from Brazil, in a collaborative study encompassing five specialized centers.

## Methods

### Design and subjects

We performed a multicenter historical cohort at five neurogenetic disorders centers in Brazil: one center from Southern and four centers from Southeastern regions. Childhood onset HSP was defined as disease onset before 12 years of age.

Index cases and affected relatives´ data were reviewed from consecutive families with clinical suspicion of HSP recruited from 2011 to 2020. Subjects were eligible if there was a suspicion of childhood-onset HSP, according to clinical diagnosis criteria^5,9^, and the diagnosis was confirmed by genetic testing. However, consecutive patients with unsolved genetic diagnosis were also included at two of the five centers, when there was a negative diagnostic workup evaluation that included a next generation sequencing panel with 12 genes related to the most frequent genes related to HSP (for full description see reference^6^) or exome sequencing (ES). These cases were included to assess the overall frequency of HSPs and the yield of contemporary clinical practice genetic investigation (See the study flowchart on Supplemental Fig. 1 for details). Patients with adolescent or adult onset, undefined age at onset or that were diagnosis with an acquired disease or with another neurogenetic disease in which the pyramidal findings were not the main clinical syndrome were excluded from the study. The study was approved by the Ethics in Research Committee—Comitê de Ética em Pesquisa do Hospital de Clínicas de Porto Alegre (GPPG-HCPA/14-0695; 62653816.7.0000.5404), which follows the Declaration of Helsinki. Informed written consent was obtained from individuals' or their guardians or data utilization consent was signed for reviewing medical records.

**Figure 1:**
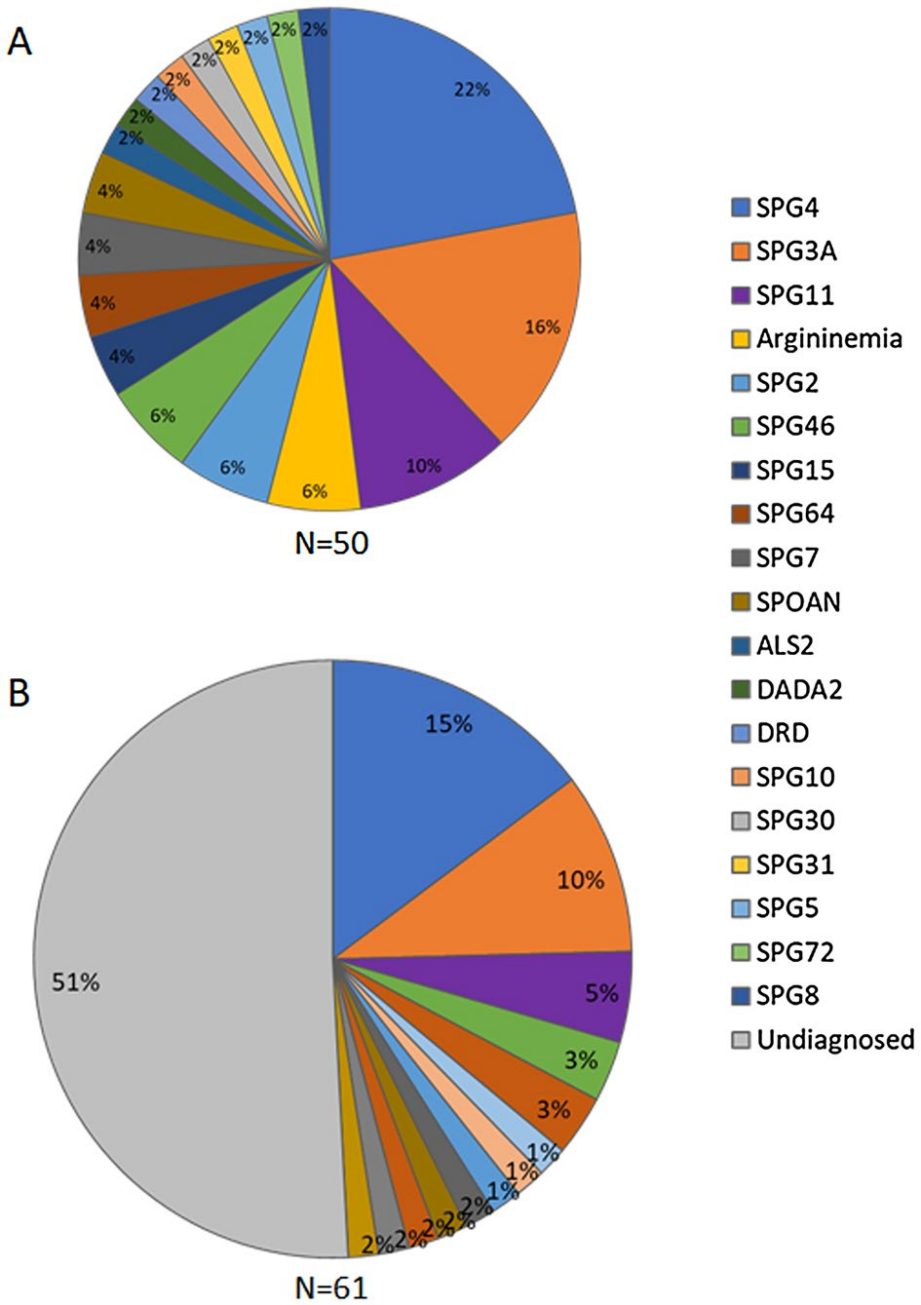
Relative frequencies of childhood-onset HSP in Brazil. Relative frequencies of childhood-onset HSP subtypes in Brazil with solved genetic diagnosis (52 probands, **A**) and of patients with childhood-onset HSP suspicion, including patients with unsolved genetic diagnosis, with data from two centers (62 probands, **B**) is presented in percentages. *DADA2* adenosine deaminase 2 deficiency, *DRD* dopa-responsive dystonia, *HSP* hereditary spastic paraplegia, *SPOAN* spastic paraplegia, optic atrophy, and neuropathy.

### Neurological and genetic evaluation

We collected data regarding sex, age, parental consanguinity, family recurrence, age at onset (first motor sign), disease duration, disease duration at walking aid (DDWA) and wheelchair (DDWC) dependency. Such information was reviewed from medical records, being confirmed by patients and relatives when necessary. We also collected information on disease severity with the Brazilian Portuguese version of Spastic Paraplegia Rating Scale (SPRS, range: 0–52, crescent in severity)^12^. Cross-sectional progression per year was assessed by dividing the last SPRS score by the disease duration at that moment, when available. HSP clinical form and frequency of complicating features defined by the neurological examination and anamnesis were also reviewed in medical records, which included information about intellectual disability, ataxia, extrapyramidal symptoms, dysphagia, dysarthria, peripheral neuropathy, lower motor neuron disease and deformities; and magnetic resonance imaging (MRI) findings, when available.

DNA was extracted from peripheral blood. A single center performed a targeted NGS panel including 12 HSP-related genes *ATL1, BSCL2, CYP27A1CYP7B1, KIAA0196, KIF5A, NIPA1, REEP1, SPAST, SPG7, SPG11* and *ZFYVE26* and the remaining centers, performed ES or target Sanger sequencing of *SPAST*. Sequences were searched for using the National Center for Biotechnology Information (NCBI) protein database. Sequence variations were compared to data available in the Human Gene Mutation Database (HGMD) and ClinVar. Variants were classified according to the 2015 American College of Medical Genetics and Genomics (ACMG)/Association for Molecular Pathology (AMP) criteria^13^ and the updated recommendations for PVS1^14^ and PP5^15^. PolyPhen-2^16^, SIFT^17^, CADD^18^, M-CAP^19^, REVEL^20^, Mutation-Taster^21^ were used for in silico analysis. Phylogenetic conservation was estimated with Genomic Evolutionary Rate Profiling (GERP + +)^22^, and allele frequencies were searched on gnomAD^23^.

### Statistical analysis

Normal distribution was evaluated by Shapiro–Wilk test. Quantitative features were reported as mean and standard deviation (SD) for parametric and median and interquartile range (IQR) for non-parametric data. Median and IQR of DDWA or DDWC were calculated by Kaplan–Meier survival analysis.

## Results

We included 106 patients (from 83 families) with suspicion of childhood-onset HSP, being 68 (from 50 families) with solved genetic diagnosis, 6 (from 5 families) with candidate variants in HSP-related genes and 32 (28 families) with unsolved genetic diagnosis. Pure HSP phenotype was present in 55/101 (54.4%) individuals in the overall cohort, in 39/68 (57.3%) individuals with solved genetic diagnosis and in 23/50 (46%) probands with solved genetic diagnosis.

### Pure-HSP with solved diagnosis

19/24 (79.1%) index cases were classified as AD-HSP, 2/24 (8.3%) as AR-HSP and 1/24 (4.2%) was an isolated case; mean (SD) age at onset was 2.98 (3.07) years, disease duration 23.2 (15.8) years, SPRS 17.7 (10.2) points and SPRS cross-sectional disease progression 1.11 (1.16) points/year (available from 13/24 cases); 9/24 (37.5%) individuals with pure HSP required walking aids and 3/23 (13%) were wheelchair dependent.

### Complex-HSP with solved diagnosis

18/26 (69.2%) index cases were classified as AR-HSP, 2/26 (7.7%) as AD-HSP, 3/26 (11.5%) as X-linked HSP and 1/26 (3.8%) was an isolated case; mean (SD) age at onset was 4.81 (3) years, disease duration 26 (11.9) years, SPRS 27.8 (13.7) points and SPRS cross-sectional disease progression 1.12 (0.53) points/year (available from 10/26 cases); 18/25 (72%) individuals with complex HSP required walking aids and 14/25 (56%) were wheelchair dependent.

### Relative frequencies of childhood onset HSP

The most common childhood onset HSP subtype in our cohort was SPG4, comprising 11/50 (22%) families with solved genetic diagnosis; followed by SPG3A, 8/50 families (16%); SPG11 in 5 families (10%) and SPG46 in 3 families with solved diagnosis and two families with bi-allelic candidate variants in *GBA2* (Fig. 1A). We were also able to report the overall frequency of subtypes in patients with HSP suspicion, including patients with unsolved genetic diagnosis, with data from two centers, one from southern and one from southeastern region of Brazil, which reported in total 61 index cases (79 individuals). In this sample 31/61 (50.8%) of index cases remained with unsolved diagnosis, but in 3/31 we identified strong candidate variants in HSP related genes (Supp. Table 1). SPG4 was the most frequent childhood onset HSP subtype, representing 9/61 (14.75%) of childhood onset HSP and 9/20 (30%) of childhood onset HSP with solved genetics diagnosis, followed by SPG3A as the second most common subtype, see Fig. 1B.

### Most frequent childhood-onset HSPs

**Figure 2:**
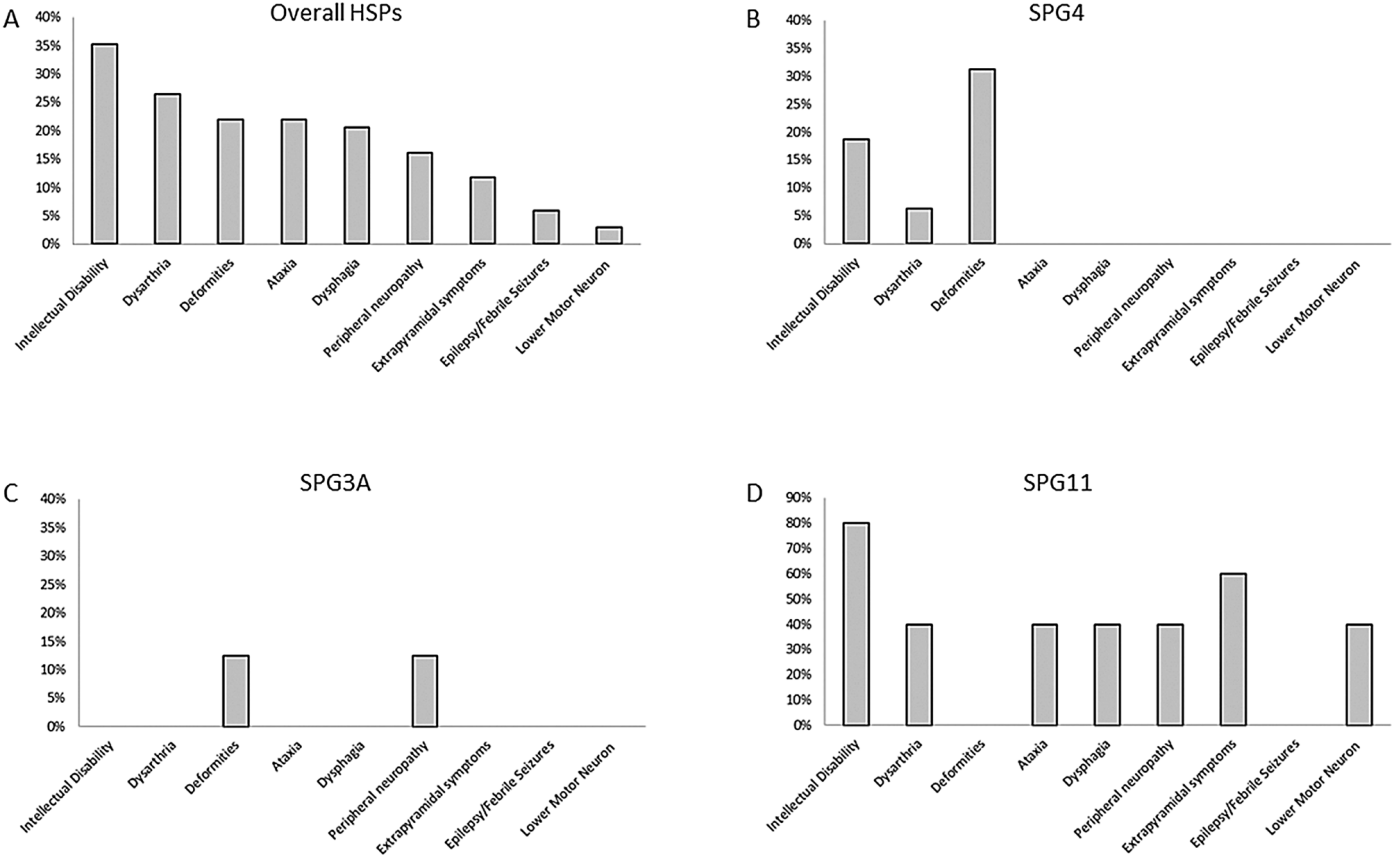
Most frequent complicating features of childhood-onset HSP. Most frequent complicating features, presented in percentages, in the overall childhood-onset HSP subjects **(A)**, in SPG4 **(B)**, in SPG3A **(C)** and in SPG11 **(D)**. *HSP* hereditary spastic paraplegia.

Table 1 depict clinical and demographical characteristic of the most frequent HSP subtypes in the present cohort. Figure 2 depicts the frequencies of complicating features in patients with solved diagnosis and Supplemental Table 1 all HSP subtypes identified in this study.

**Table 1:** Clinical characteristics of most frequent childhood-onset HSP subtypes.

	SPG4	SPG3A	SPG11	SPG46
Gene	*SPAST*	*ALT1*	*SPG11*	*GBA2*
Patients (families)	16 (11)	16 (8)	5 (5)	5 (5)
Male sex (%)	43.8%	50%	80%	40%
Pure form (%)	100%	87.5%	0%	0%
Age (years)	21.1 (13.07)	24.8 (15.2)	27.6 (10.1)	40.4 (10)
Age at onset (years)	3 (3.09)	2.2 (2.54)	7.2 (1.79)	7.8 (2.3)
Disease duration (years)	18.2 (12.4)	23.4 (15.1)	20.4 (10.4)	32.6 (11.4)
Walking aid (%)	4/16 (25%)	8/16 (50%)	4/5 (80%)	3/4 (75%)
DDWA (years)	NA	36 (4 to NA)	11 (11 to 25)	18 (5 to 20)
Wheelchair bound (%)	1/16 (6.2%)	1/16 (6.2%)	4/5 (80%)	1/4 (25%)
DDWC (years)	NA	NA	23 (5 to 25)	NA
SPRS^a^	12 (8.3)	20.3 (10.6)	24.3 (18.5)	NA
SPRS/disease duration	1.08 (0.87)	1.14 (1.32)	1.37 (0.57)	NA

Data are shown as mean and standard deviation, except for DDWA and DDWC in which data are shown as median and interquartile ranges.

*DDWA* disease duration at walking aid dependency, *DDWC* disease duration at wheelchair dependency, *HSP* hereditary spastic paraplegias, *SPRS* spastic paraplegia rating scale.

^a^Available for 11 cases with SPG4, 10 cases with SPG3A and 3 cases with SPG11.

### SPG 4

**Figure 3:**
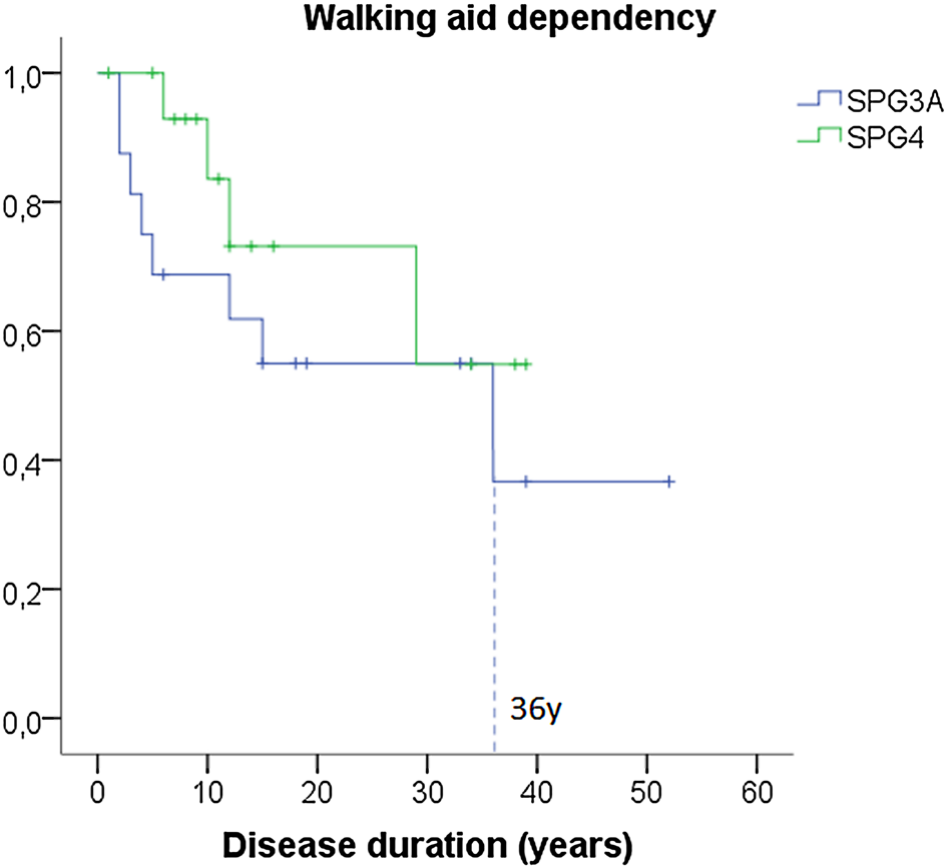
Progression to major handicap in SPG4 and SPG3A. The figure shows Kaplan–Meier analysis of loss of independent walking to disease duration for individuals with SPG4 and SPG3A. Median values of any walking aid assistance for SPG3A is informed and highlighted by dashed lines.

The most common childhood onset HSP in our cohort was SPG4, in which eleven families with 16 individuals in total were described. Missense variants were observed in 6/11 (54.5%) families, nonsense variants in 2/11, and frameshift, canonical splice site and intronic variants in 1 family each. Figure 3 shows the survival curves for walking device assistance in SPG4. Because only 25% of individuals required walking aid the median DDWA was not achieved for SPG4. A single SPG4 subject was wheelchair bound. Cross-sectional SPRS disease progression (available for 11/16 patients) was 1.08 (0.87) points per year of disease. Three SPG4 patients from a single family presented mild intellectual disability, one of them with mild dysarthria. Five patients presented foot deformities (3 families, Fig. 2B). Brain MRI was available for 7 cases, one presenting a small pineal cyst and the others normal scans.

### SPG3A

The second most common childhood onset HSP in our cohort was SPG3A, in which eight families with 16 individuals in total were described. Missense variants were observed in all families. One SPG3A subject presented complex HSP. Figure 3 shows the survival curves for walking device assistance, with a median DDWA of 36 years. A single SPG3A subject was wheelchair bound. Cross-sectional SPRS disease progression (available for 10/16 patients) was 1.14 (1.83) points per year of disease. Frequencies of complicating features are presented in Fig. 2C. Brain MRI was available for 4 cases, none with abnormal scans.

### SPG11

Five patients (5 families) with SPG11 were reported, all of them with complex HSP. All patients presented loss of function pathogenic variants, 2 with canonical splice site, 2 with frameshift and 1 with nonsense variants. Only a single SPG11 patient did not require walking aid assistance. Median DDWA was 11 years and median DDWC was 23 years; however, there was one missing data for age at wheelchair dependency, in which we have imputed the same values of DDWA (conservative bias) in order to avoid that DDWC would be lower than DDWA. Cross-sectional SPRS disease progression was 1.37 (0.57) points per year of disease (available for 3/5 patients). Frequencies of complicating features are presented in Fig. 2D. Brain MRI of the five SPG11 patients presented thin corpus callosum and the ears of lynx sign and one individual presented unspecified leukoencephalopathy.

### SPG46

Five patients from three families with solved and two families with bi-allelic candidate variants in *GBA2* were reported, all of them with complex HSP. Walking aid assistance was required for 75% of individuals and 25% were wheelchair bound. Median DDWA was 18 years and median DDWC was not achieved. Cross-sectional SPRS disease progression was available for a single patient, being 0.66 points per year of disease. Brain MRI was available for three cases, none with abnormal scans.

### Argininemia

Argininemia, a potentially treatable rare urea cycle disorder, was diagnosed in four patients from four different families by exome sequencing, three of them homozygous for *ARG1* p.(Thr143Ile) pathogenic variant. All subject with argininemia presented a complex HSP phenotype and required walking aid assistance, and 75% were wheelchair bound. Cross-sectional SPRS disease progression was 1.04 (0.15) points per year of disease (available for 3/4 patients). Cognitive decline and ataxia were present in all cases and epilepsy in one. One subject presented normal Brain MRI and one thin corpus callosum.

### Novel variants

We have described 20 variants not previously reported in the literature in HSP-related genes (Table 2). All variants found in the present study are described in Supplemental Table 1.

**Table 2:** Novel variants in muscular HSP-related genes.

Gene (transcript)	Nucleotide/AA change	Variant type	AF^a^	SIFT	Poly Phen2	MT	MCAP	CADD	REVEL	GERP + + ^b^	Segregation	Fam	N^c^	CLINVAR	Classification	ACMG criteria^d^
*ALS2* *NM_020919.4*	c.326delA p.(Asn109Metfs*17)	Frameshift	0	NA	NA	1.000	NA	NA	NA	2.85	NA	1	1	No	Likely pathogenic	PVS1, PM2
*ATL1* *NM_015915.4*	c.574C > G p.(Leu192Val)	Missense	0	0	0.459	0.999	0.156	25.3	0.678	4.93	Yes	1	2	No	Likely pathogenic	PM1, PM2, PM5, PP1, PP2, PP3
*ATL1* *NM_015915.4*	c.829G > C (p.Ala277Pro)	Missense	0	0	0.955	0.999	0.134	23.8	0.719	4.74	NA	1	1	No	Likely pathogenic	PM1, PM2, PP2, PP3
*ATL1* *NM_015915.4*	c.1239 T > G p.(Phe413Leu)	Missense	0	0.09	0.117	0.999	0.08	19.96	0.674	−0.334	Yes	1	3	No	Likely pathogenic	PM1, PM2, PM5, PP1,PP2, PP3
*CYP7B1* *NM_004820.5*	c.650 T > A p.(Leu217*)	Nonsense	0	NA	NA	0.999	NA	33	NA	1.82	NA	1	1	No	Likely pathogenic	PVS1, PM2
*CYP7B1* *NM_004820.5*	c.887A > C p.(Asn296Thr)	Missense	0	0	1	0.999	0.018	26.1	0.538	5.93	NA	1	1	No	Likely pathogenic	PM1, PM2, PP2, PP3
*ENTPD1* *NM_001164178.1*	c.610-8_610-5delTCTT	Intronic	0	NA	NA	1.000	NA	NA	NA	3.97 (2.45)	Yes	1	2	No^f^	VUS	PM2, PP1, PP3
*ENTPD1* *NM_001164178.1*	c.806_807delGG p.(Gly269Glufs*18)	Frameshift	0	NA	NA	1.000	NA	NA	NA	5.1 (0.63)	NA	2	3^g^	No^f^	Likely pathogenic	PVS1, PM2
*GBA2* *NM_020944.3*	c.1365G > C p.(Trp455Cys)	Missense	0.00000398	0	1	0.999	0.156	32	0.715	5.6	NA	4	4	No	Likely pathogenic	PS4, PM2, PP3, BP1
*GBA2* *NM_020944.3*	c.1789G > T p.(Asp597Tyr)	Missense	0	0.01	0.997	0.999	0.014	32	0.737	5.69	NA	2	2	No	VUS	PM2,PM3,PP3,BP1
*GBA2* *NM_020944.3*	c.1943_1944delGT p.(Cys648Serfs*7)	Frameshift	0	NA	NA	1.000	NA	NA	NA	4.31 (1.22)	NA	1	1	No	Likely pathogenic	PVS1, PM2
*KIF1A* *NM_001244008.2*	c.153C > G p.(Ser51Arg)	Missense	0	0.02	0.572	0.999	0.096	25.6	0.477	2.64	NA	1	1	No	Likely pathogenic	PM1, PM2, PP2, PP3
*KIF5A* *NM_004984.4*	c.1022A > G (p.Gln341Arg)	Missense	0	0	0.413	0.999	0.116	23.6	0.554	4.38	NA	1	1	No	Likely pathogenic	PM1, PM2, PP2, PP3
*PLP1* *NM_000533.5*	c.617 T > C p.(Met206Thr)	Missense	0	0.01	0.446	0.990	0.941	24.3	0.804	5.58	NA	1	1	No	Likely pathogenic	PM1, PM2, PM5, PP2, PP3, PP4
*REEP1* *NM_022912.3*	c.55C > A p.(Pro19Thr)	Missense	0	0.04	0.247	0.999	0.129	24.6	0.552	4.35	NA	1	1	No	Likely pathogenic	PM1, PM2, PM5, PP2, PP3
*REEP2 NM_001271803.2*	c.741dupC/ × 1 p.(Ser248Leufs*39)	Frameshift	0	NA	NA	0.999^e^	NA	NA	NA	−1.44 (0.46)	Yes	1	4	No	Pathogenic	PVS1, PM2, PP1
*SPAST* *NM_014946.4*	c.1255G > T p.(Gly419*)	Nonsense	0	NA	NA	1.000	NA	42	NA	5.62	NA	1	1	No	Likely pathogenic	PVS1, PM2
*SPG7* *NM_003119.4*	c.1997G > T p.(Gly666Val)	Missense	0	0	1	0.999	0.833	28.6	0.976	5.21	NA	1	1	No^f^	Likely pathogenic	PM2, PM5, PP2, PP3
*SPG11* *NM_025137.4*	c.5490delT p.(Glu1831Asnfs*7)	Frameshift	0	NA	NA	1.000	NA	NA	NA	−1.05	NA	1	1	No	Likely pathogenic	PVS1, PM2
*ZFYVE26* *NM_015346.4*	c.3642_3643insCCACACTTAG p.(Ala1215Profs*22)	Frameshift	0.00000401	NA	NA	1.000	NA	NA	NA	3.79 (0.03)	NA	1	1	No	Likely pathogenic	PVS1, PM2

Allele frequencies on ^a^gnomAD; ^b^GERP +  + data is shown as mean (standard deviation) or raw value; ^c^total number of individuals in which the variant was detected; ^d^American College of Medical Genetics and Genomics criteria, Richards et al., 2015; ^e^polymorphism prediction; ^f^ClinVar descriptions are related to the reported cases; ^g^Two patients with genetic diagnosis and one deceased sibling with clinical diagnosis.

*AA* amino acid, *AF* allele frequency, *Fam* families, *MT* mutation taster, *NA* not available, *VUS* variant of unknown significance.

## Discussion

In the present study, we have comprehensively described clinical and molecular data of a large Brazilian cohort of patients with childhood-onset HSP. Our study indicates that SPG4, closely followed by SPG3A, is the most frequent childhood-onset subtype in Brazil. We have described 20 novel variants in HSP-related genes and provided the progression to major handicap of the most frequent subtypes in our cohort.

A slightly predominance of complex forms (52%) was found among probands with childhood-onset HSP, which is similar to previous studies^8^. A greater frequency of complex forms in childhood-onset HSP was previously reported in smaller sample studies that evaluated individuals from populations with high consanguinity rates^24,25^, and also in another study carried out in Brazil that showed a prevalence rate of complex forms of 65.8% among 20 individuals with childhood-onset HSP suspicion, but without molecular diagnosis confirmation^26^.

SPG4 was the most frequent childhood-onset HSP in the present study, representing 22% of probands with solved genetic diagnosis, followed by SPG3A, representing 16% of probands. There has been controversy about the most frequent HSP subtype with childhood-onset. While some reports indicated SPG3A as the most frequent subtype, with frequencies varying between 36 and 75% of patients^7–9^; others reported SPG4 as the most common childhood-onset subtype^4,9,10^. Worldwide data suggest that both SPG4 and SPG3A are the most common childhood-onset HSPs and the predominance of one subtype over another in the different countries might be explained by different population genetic background and sampling biases.

One of the core explanations for families with childhood-onset SPG4 is genotype–phenotype correlation, implicating missense variants to earlier onset^27,28^. A very large sample study that evaluated 842 SPG4 individuals most from France reported that missense variants were related to 10 years earlier ages at onset when compared to truncating variants in *SPAST,* and that most subjects with pathogenic missense variants in* SPAST* had disease onsets before the second decade of life^29^. We found that 54.5% of probands with childhood-onset SPG4 carried missense pathogenic variants in *SPAST*, a greater proportion than the 33% (87/266) of missense variants reported by the French study for the overall SPG4 probands^29^. It was also a greater proportion than what was reported in recent Portuguese and Russian studies, which reported missense variants being responsible for 11/27 (40%) and 10/31 (32.2%) of SPG4 probands regardless of the age at onset, respectively^28,30^. Our results favor the concept that missense variants are associated to earlier ages at onset of SPG4, since our sample was exclusively of childhood-onset HSP subjects. Future studies covering the entire HSP population across all age groups in Brazil will be able to perform further analysis on this genotype–phenotype correlation.

Although missense variants are related to earlier ages at onset in SPG4, 45.5% of childhood-onset SPG4 in our study were not related to this type of variant, indicating that additional factors should also explain earlier onset in this disease. The study by Parodi and collaborators^29^ showed that the intragenic polymorphism in *SPAST* p.(Ser44Leu) was associated with earlier onset, starting on average 11 years earlier than subjects that did not carry the polymorphism. However, as the *SPAST* p.(Ser44Leu) polymorphism was present in only 11/558 cases in that study, it does explain only a little proportion of families with childhood-onset SPG4. On the other hand, the effect of this polymorphism in the age of onset of a small subpopulation of SPG4 individuals, indicates that other polymorphisms, intragenic or not, might be capable of modulating the disease onset. Among the 3/11 SPG4 probands with childhood onset of the present study in which this information was available, none carried the *SPAST* p.(Ser44Leu) polymorphism.

Missense variants around the GTPase binding domain were previously associated to earlier ages at onset in SPG3A, while frameshift variants in the C-terminus of atlastin-1 were associated to later disease onset^31^. All childhood-onset subjects with SPG3A in our study carried missense variants, supporting this concept. However, considering that SPG3A is caused mainly by missense variants^28,30^ and that it usually starts during childhood it is harder to establish this genotype–phenotype correlation. A toxic gain of function related to atlastin-1 modification by missense variants would be a plausible explanation for this disease being rarely caused by truncated variants; however, data from Genome Aggregation Database (gnomAD) indicate that this gene does not tolerate loss-of function with a pLI score of 0.98^23^. Therefore, it is not clear whether haplo-insufficiency, dominant negative effects or toxic gain of function are the main molecular mechanism of the disease. Future studies covering the entire HSP population across all age groups in Brazil might be able to perform further analysis on this genotype–phenotype correlation for SPG3A.

### Pure-HSP

Twenty-five percent of subjects with SPG4 (after mean disease duration of 18 years) and 50% of subjects with SPG3A (after a mean disease duration of 23.4 years) required some kind of walking aid assistance. Median disease duration at walking aid assistance (DDWA) was just possible to calculate for SPG3A and it occurred 36 years after disease onset (Fig. 3). The cross-sectional progression of 1.08 and 1.14 points per year for SPG4 and SPG3A, respectively, is compatible with a very slow disease progression, although longitudinal data from future studies are needed to better establish SPRS progressions in these diseases.

### Complex-HSP

SPG11 and SPG46 were the most common subtypes of complex HSPs with childhood onset in the present study. Similar to previous studies, although presenting later onset than pure forms, complex HSPs presented faster disease progressions with greater handicap, requiring canes or wheelchair assistance earlier than SPG3A and SPG4, with 80% of SPG11 and 25% of SPG46 cases being wheelchair bound. Due to the low number of subjects analyzed for cross-sectional SPRS progressions with SPG11 and SPG46, the results presented in Table 1 should be interpreted with caution. Further larger sample studies will need to address SPRS progression for these subtypes.

The most common complicating features of childhood-onset HSP with solved genetic diagnosis in our series was cognitive impairment/intellectual disability, which was present in 35% of subjects; followed by dysarthria, present int 26% of cases, joint deformities (mostly feet deformities), present in 22% of cases; ataxia and dysphagia, present in 22 and 21% of cases, respectively; and by peripheral neuropathy, present in 16% of cases. Similar to our findings, some studies have reported cognitive impairment/intellectual disability as the most common complicating feature of childhood-onset HSP, varying from 23^24^ to 87%^26^. Of note, three SPG4 patients from a single family presented mild intellectual disability, which was only perceived during formal cognitive evaluations, presenting mild, but abnormal cognitive performances^32^.

Qualitative findings on brain MRI have been evaluated in the present study with available data for 58 subjects, 42 of them with solved genetic diagnosis, in which 16/42 (38.1%) presented abnormal findings. The most frequent MRI abnormality was thin *corpus callosum* (TCC), which was present in 10/42 subjects (5 with SPG11, 2 with SPG15, 1 each with argininemia, SPG30 and SPG64). In 8/10 of subjects with TCC it was also described the “ears of the lynx '' sign, which was not reported only in the subject with argininemia and SPG64. Other relevant MRI features were brain atrophy and leukoencephalopathy, findings that were detailed in Supplemental Table 1. MRI findings of the present study are similar to the literature^33^.

### Study limitations

Our study represents one of the largest reported cohorts with comprehensive clinical and molecular data on childhood-onset HSP reported so far. However, it has several limitations. Genetic investigation protocols and genetic testing availability was not homogenous across centers and therefore we were only able to include consecutive cases with confirmed genetic diagnosis and unsolved cases in 2/5 centers, limiting the information regarding the proportion of unsolved cases after performing targeted panel of genes or ES. Nevertheless, the classification of genetic variants was standardized across centers and all variants were reviewed and classified by the same medical geneticists (JAMS) with posterior validation of the study group. Another limitation is that specific copy number variation analysis for *SPAST* or *ATL1* was nor performed and relative frequencies of these subtypes may have been underestimated. We have calculated cross-sectional SPRS disease progressions for SPG4, SPG3A and SPG11; which are valuable information considering the lack of clinical course and natural history data on validated instruments for HSP; however, future longitudinal studies are required to establish progression rates in HSP in a more reliable way and with lower chance of recall bias related to information regarding age at onset and disease duration. We had significant missing data for some variables, for instance, we only have brain MRI information for 58/106 subjects. Because all cases were recruited at specialized neurogenetics centers in teaching hospital, relative frequencies of HSP in Brazil based on population-based studies might be different than ours.

## Conclusion

In conclusion, in this large-scale historical cohort study, we were able to define SPG4 as the most frequent childhood-onset subtype of HSP in Brazil, closely followed by SPG3A and to reinforce the finding relating missense variants in *SPAST* to earlier onsets of SPG4. Probands presenting with complex forms were slightly more common than probands with pure forms in the childhood population. Cognitive impairment/intellectual disability was the most common complicating feature, followed by joint deformities, ataxia, dysarthria, peripheral neuropathy and dysphagia, which, with the exception of joint deformities, mostly presented in complex forms. Our data confirmed the slow progression to handicap for SPG4 and SPG3A, which was in accordance with the slow cross-sectional SPRS progression for these subtypes. These results are paramount to understand the epidemiology of early-onset HSP in Brazil, as well as to understand better these disease progressions.

## Supplementary Information

Supplementary Figure S1.

Supplementary Table S1.

Supplementary Information 1.

Supplementary Information 2.

## Data Availability

The data that support the findings of this study are available from the corresponding author upon reasonable request.
