# Supplementary figures and images for: Clinical and molecular characterization of a large cohort of childhood onset hereditary spastic paraplegias
Source: Sci Rep. 2021 Nov 15;11:22248. doi: 10.1038/s41598-021-01635-2 (PMC8593146; doi:10.1038/s41598-021-01635-2)

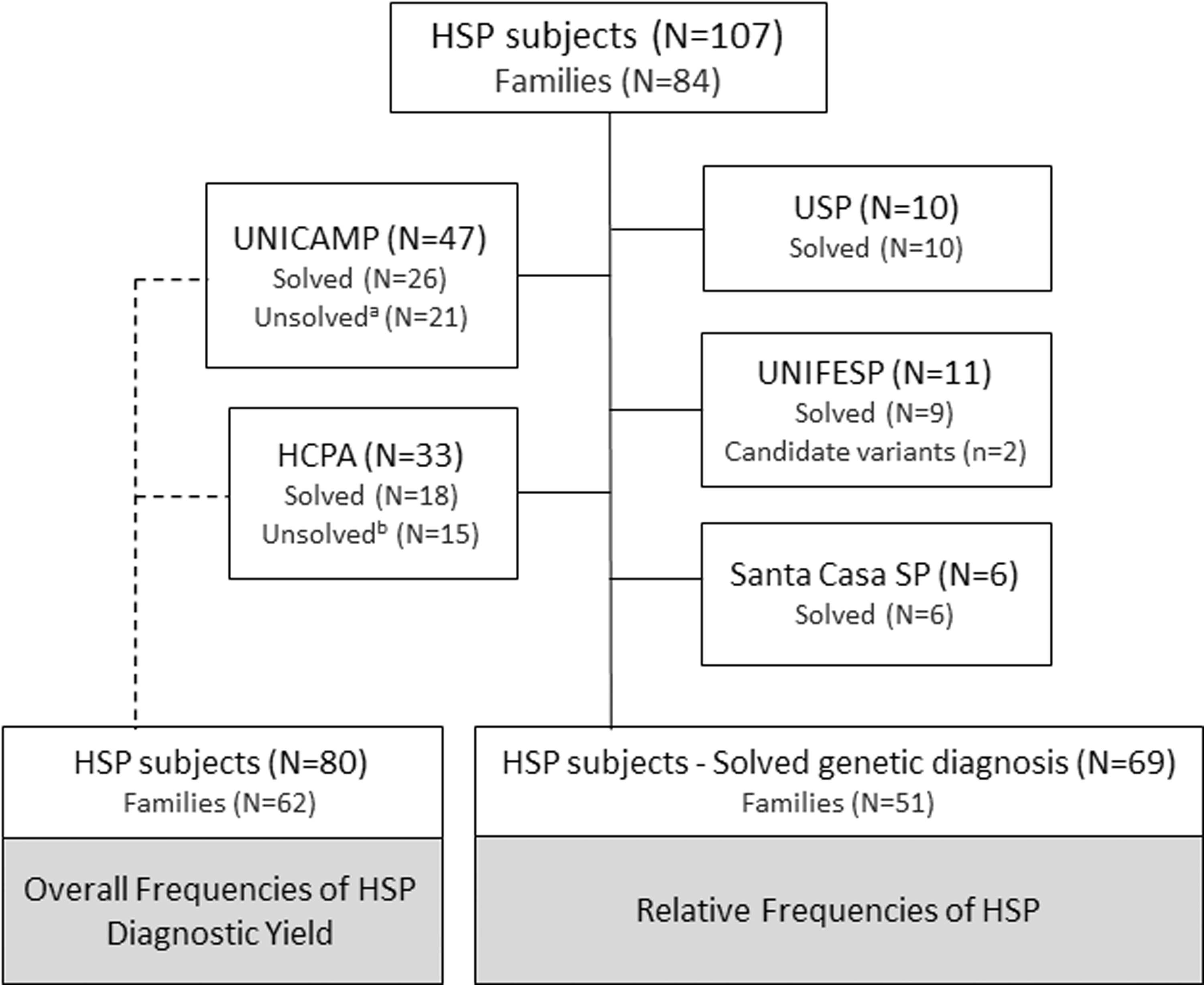

Supplement: Supplementary file 1 — Supplementary Figure S1. [file 41598_2021_1635_MOESM1_ESM.tif]
