# Supplementary material for: Clinical and molecular characterization of a large cohort of childhood onset hereditary spastic paraplegias
Source: Sci Rep. 2021 Nov 15;11:22248. doi: 10.1038/s41598-021-01635-2 (PMC8593146; doi:10.1038/s41598-021-01635-2)
Supplement: Supplementary file 3 — Supplementary Information 1. [file 41598_2021_1635_MOESM3_ESM.docx]

**Supplemental Figure 1: Flowchart of the study**

Relative frequencies of HSP subtypes were provided for cases with solved genetic diagnosis from all five centers (full lines). Overall frequencies including cases with unsolved genetic diagnosis were provided with data from two centers HCPA and UNICAMP (dashed lines). HCPA center is located in southern Brazil and the other centers in Southeastern region. ^a^Minimal investigation for HSP cases to be considered unsolved was a negative result on a NGS panel of 12-HSP related genes; ^b^Minimal investigation for HSP cases to be considered unsolved was a negative result on exome sequencing. HSP, hereditary spastic paraplegia.
