# Supplementary material for: Clinical and molecular characterization of a large cohort of childhood onset hereditary spastic paraplegias
Source: Sci Rep. 2021 Nov 15;11:22248. doi: 10.1038/s41598-021-01635-2 (PMC8593146; doi:10.1038/s41598-021-01635-2)
Supplement: Supplementary file 4 — Supplementary Information 2. [file 41598_2021_1635_MOESM4_ESM.docx]

**Supplemental Table 1 – Detailed clinical and genetic information of childhood-onset HSP individuals**

AA, Amino acid; ACMG, American College of Medical Genetics and Genomics; AD, Autosomal Dominant; AO, Age at Onset; AR, Autosomal Recessive; DD, Disease Duration; DDWA, Disease Duration at Walking Aid dependency; DDWC, Disease Duration at Wheelchair dependency; ES, exome sequencing; HSP, Hereditary Spastic Paraplegia; LMN, Lower Motor Neuron; MRI, Magnetic Resonance Imaging; PNP, Peripheral Neuropathy; SPRS, Spastic Paraplegia Rating Scale; TCC, Thin corpus callosum; ^1^NGS panel - 104 inherited neuropathies related genes; ^2^NGS panel including *GCH1, SPR, TH*.
